# Supplementary material for: Saccharomyces boulardii (CNCM I-745) improves intestinal damage in sepsis by remodeling intestinal flora structure
Source: Front Cell Infect Microbiol. 2025 Jul 31;15:1602792. doi: 10.3389/fcimb.2025.1602792 (PMC12350329; doi:10.3389/fcimb.2025.1602792)
Supplement: Supplementary file 1 [file DataSheet1.zip › summary/summary/8_Advanced_analyse/2_PICRUSt2_result/KEGG/Group.CW4_vs_CW0/PICRUSt2_KO_Group.CW4_vs_CW0_diff.pdf]

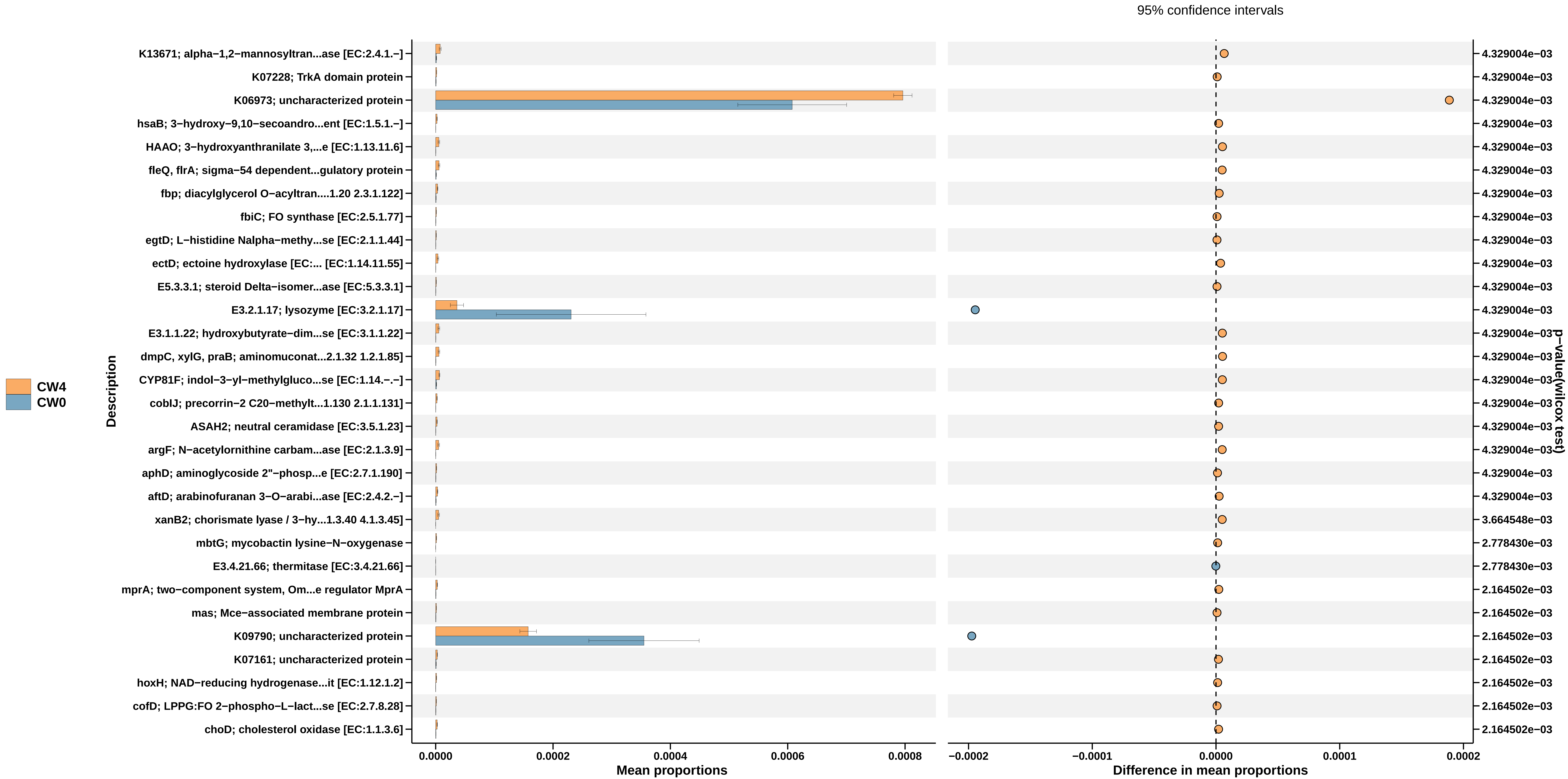
95% confidence intervals
K13671; alpha−1,2−mannosyltran...ase [EC:2.4.1.−]
K07228; TrkA domain protein
K06973; uncharacterized protein
hsaB; 3−hydroxy−9,10−secoandro...ent [EC:1.5.1.−]
HAAO; 3−hydroxyanthranilate 3,...e [EC:1.13.11.6]
fleQ, flrA; sigma−54 dependent...gulatory protein
fbp; diacylglycerol O−acyltran....1.20 2.3.1.122]
fbiC; FO synthase [EC:2.5.1.77]
egtD; L−histidine Nalpha−methy...se [EC:2.1.1.44]
ectD; ectoine hydroxylase [EC:... [EC:1.14.11.55]
E5.3.3.1; steroid Delta−isomer...ase [EC:5.3.3.1]
E3.2.1.17; lysozyme [EC:3.2.1.17]
E3.1.1.22; hydroxybutyrate−dim...se [EC:3.1.1.22]
dmpC, xylG, praB; aminomuconat...2.1.32 1.2.1.85]
CYP81F; indol−3−yl−methylgluco...se [EC:1.14.−.−]
cobIJ; precorrin−2 C20−methylt...1.130 2.1.1.131]
ASAH2; neutral ceramidase [EC:3.5.1.23]
argF; N−acetylornithine carbam...ase [EC:2.1.3.9]
aphD; aminoglycoside 2"−phosp...e [EC:2.7.1.190]
aftD; arabinofuranan 3−O−arabi...ase [EC:2.4.2.−]
xanB2; chorismate lyase / 3−hy...1.3.40 4.1.3.45]
mbtG; mycobactin lysine−N−oxygenase
E3.4.21.66; thermitase [EC:3.4.21.66]
mprA; two−component system, Om...e regulator MprA
mas; Mce−associated membrane protein
K09790; uncharacterized protein
K07161; uncharacterized protein
hoxH; NAD−reducing hydrogenase...it [EC:1.12.1.2]
cofD; LPPG:FO 2−phospho−L−lact...se [EC:2.7.8.28]
choD; cholesterol oxidase [EC:1.1.3.6]
Description
CW4
CW0
0.0000
0.0002
0.0004
0.0006
0.0008
Mean proportions
−0.0002
−0.0001
0.0000
0.0001
0.0002
Difference in mean proportions
p−value(wilcox test)
4.329004e−03
4.329004e−03
4.329004e−03
4.329004e−03
4.329004e−03
4.329004e−03
4.329004e−03
4.329004e−03
4.329004e−03
4.329004e−03
4.329004e−03
4.329004e−03
4.329004e−03
4.329004e−03
4.329004e−03
4.329004e−03
4.329004e−03
4.329004e−03
4.329004e−03
4.329004e−03
3.664548e−03
2.778430e−03
2.778430e−03
2.164502e−03
2.164502e−03
2.164502e−03
2.164502e−03
2.164502e−03
2.164502e−03
2.164502e−03
